# The Parkinson’s disease-associated LRRK2-G2019S variant restricts serine metabolism, leading to microglial inflammation and dopaminergic neuron degeneration

**DOI:** 10.1186/s12974-025-03577-2

**Published:** 2025-10-27

**Authors:** Henry Kurniawan, Sarah L. Nickels, Alise Zagare, Elisa Zuccoli, Isabel Rosety, Gemma Gomez-Giro, Enrico Glaab, Jens C. Schwamborn

**Affiliations:** 1 Developmental and Cellular Biology, Luxembourg Centre for Systems Biomedicine (LCSB), University of Luxembourg, Esch-Sur-Alzette, Luxembourg; 2 Biomedical Data Science Group, Luxembourg Centre for Systems Biomedicine, University of Luxembourg, Esch-Sur-Alzette, Luxembourg

**Keywords:** LRRK2-G2019S, Microglia, Parkinson’s disease, Metabolism, Serine, Glycolysis, IPSC, Organoids

## Abstract

A growing body of evidence implicates inflammation as a key hallmark in the pathophysiology of Parkinson’s disease (PD), with microglia playing a central role in mediating neuroinflammatory signaling in the brain. However, the molecular mechanisms linking microglial activation to dopaminergic neuron degeneration remain poorly understood. In this study, we investigated the contribution of the PD-associated LRRK2-G2019S mutation to microglial neurotoxicity using patient-derived induced pluripotent stem cell (iPSC) models. We found that LRRK2-G2019S mutant microglia exhibited elevated activation markers, enhanced phagocytic capacity, and increased secretion of pro-inflammatory cytokines such as TNF-α. These changes were associated with metabolic dysregulation, including upregulated glycolysis and impaired serine biosynthesis. In 3D midbrain organoids, these overactivated microglia resulted in dopaminergic neuron degeneration. Notably, treating LRRK2-G2019S microglia with oxamic acid, a glycolysis inhibitor, attenuated microglial inflammation and reduced neuronal loss. Our findings underscore the link between metabolic targeting in microglia and dopaminergic neuronal loss in LRRK2-G2019S mutation, and highlight a potential strategy that warrants further preclinical evaluation.

**Supplementary Information:**

The online version contains supplementary material available at 10.1186/s12974-025-03577-2.

## Introduction

Parkinson’s disease (PD) stands as the second most common neurodegenerative disorder, with its incidence steadily rising over time [1]. While mitochondrial dysfunction, oxidative stress, calcium imbalance, and impaired autophagy and mitophagy have long been recognized as key contributors to dopaminergic neuron loss [2], neuroinflammation has emerged in recent years as a prominent and unifying feature across PD subtypes [3]. Accordingly, targeting neuroinflammatory pathways presents a promising therapeutic strategy for PD.

Microglia, the brain’s resident immune cells, play essential roles in both immune defense and neuronal homeostasis. In response to pathogen-associated molecular patterns, microglia activate and release cytokines to recruit peripheral immune cells and contain damage. Beyond immune surveillance, microglia support neuronal development by regulating neuronal progenitor cells, guiding dopaminergic axon growth, and releasing neurotrophic factors [4]. Importantly, they are also key mediators of aggregated α-synuclein clearance – another pathological hallmark of PD [5]. However, chronic engagement in α-synuclein clearance can perpetuate a cycle of inflammation, ultimately resulting in neurotoxicity and neuronal death [6, 7]. This vicious cycle is increasingly recognized as a critical driver of PD progression.

Immune cells, including microglia, shift from a resting state to an activated state upon receiving stimulatory signals, accompanied by adaptations in their metabolic pathways – a process known as metabolic reprogramming. This metabolic shift is fundamental for providing the energy required for cell proliferation, differentiation, and activation. Studies have shown that peripheral immune cells, including microglia, switch from a highly efficient ATP production via mitochondrial oxidative phosphorylation to faster ATP production through glycolysis [8]. The fine-tuning of cellular metabolism is crucial for normal immune cell functions in both health and disease [9, 10]. In addition to glucose metabolism, glucose is diverted to the pentose phosphate pathway to produce NADPH and ribose 5-phosphate, a precursor for nucleotide synthesis. Additionally, glucose can also be channeled into serine synthesis and one-carbon metabolism, supplying proteins, nucleic acids, lipids, and macromolecules to support cell proliferation and growth [11]. Microglia can also utilize free fatty acids as alternative energy sources in the absence of glucose, indicating their metabolic flexibility [12]. These metabolic dynamics highlight the importance of metabolic rewiring to provide sufficient precursors and energy to maintain normal microglial function. It has been hypothesized that during chronic inflammation in PD, microglial metabolism becomes compromised [8, 13, 14]. However, due to the complex etiology of PD, the precise link between PD pathology and microglial metabolic rewiringr remains unclear.

In this study, we examined how the LRRK2-G2019S mutation, one of the most common genetic causes of familial and sporadic PD, affects microglial physiology. Our results show that LRRK2-G2019S drives a proinflammatory phenotype in microglia by reprogramming their metabolism, notably enhancing glycolysis and impairing serine biosynthesis. These changes contribute to dopaminergic neurotoxicity in otherwise healthy midbrain organoids. Importantly, pharmacological targeting of glucose metabolism with oxamic acid ameliorated microglial activation and protected neurons. These findings are consistent with a functional connection between microglial immunometabolism and dopaminergic neuron vulnerability, and suggest avenues to explore for restoring microglial homeostasis.

## Materials and methods

### Cell cultures

#### Human induced pluripotent stem cells (iPSCs) culture

iPSCs (Table 1) were generated as previously described [15] and maintained on GelTrex™ hESC-qualified coated 6-well plates (ThermoFisher Scientific, A1569601) in Essential 8 Basal medium (ThermoFisher Scientific, A157001) supplemented with ROCK inhibitor (Y-27632, Merck Millipore, SCM075) for the first 24 h post-passaging. Medium was exchanged daily. At ~ 80% confluence, iPSCs were split dissociated using Accutase® (Sigma, A6964) and reseeded at approximately 500,000 cells per well.

**Table 1:** List of human iPSCs used in this study with details of the gender and age of sampling. The study uses four healthy and four patient-derived iPSCs

Original ID	Diagnosis	Sex	Age
DB 202	LRRK2-WT	M	55–59
DB 262	LRRK2-WT	F	57
DB 389	LRRK2-WT	M	-
DB 201	LRRK2-WT	F	-
DB 209	LRRK2-G2019S	F	81
DB 228	LRRK2-G2019S	F	40
DB 222	LRRK2-G2019S	F	66
DB 226	LRRK2-G2019S	M	79

Macrophage precursors were generated from iPSCs based on previous protocols [16]. Briefly, 4 × 10^6^ iPSCs were seeded into AggreWell™ 800 plate pre-treated with Anti-Adherence Rinsing Solution (Stemcell Technologies, 34860). Cells were cultured for 4 days in embryoid body (EB) medium, consisting of Essential 8 Basal medium supplemented with ROCK inhibitor, 50 ng/ml bone morphogenetic protein 4 (BMP4, Biolegend, 795606), 50 ng/ml vascular endothelial growth factor 165 (VEGF-165, Biolegend, 583708), and 20 ng/ml stem cell factor (SCF, Miltenyi Biotec, 130–096–695). EBs were harvested and transferred to ultra-low attachment 6-well plates (ThermoFisher Scientific, 07–200–601) and cultured for an additional 3 days in EB medium. Subsequently, EBs were transferred to a T75 flask and cultured in Factory medium (X-VIVO15 supplemented with 100 ng/ml macrophage colony-stimulating factor M-CSF, Biolegend 574808 and 25 ng/ml IL-3, Biolegend 578008) to generate macrophage precursors.

#### Microglia differentiation

Macrophage precursors were harvested biweekly and plated at either 50,000 (96-well) or 1 × 10^6^ (6-well) cells/well. Cells were cultured for 14 days in basal microglia differentiation medium composed of Advanced DMEM/F12 (ThermoFisher Scientific, 35050061), 1 × N2 (ThermoFisher Scientific, 17502001), 1 × Pen/Strep (Invitrogen, 15140122), 1 × GlutaMax (ThermoFisher Scientific, 35050061), 50 µM β-Mercaptoethanol (ThermoFisher Scientific, 31350–010) supplemented with 100 ng/ml IL-34 (Biolegend, 577906), and 10 ng/ml GM-CSF (Biolegend, 572905) as described previously [17]. Medium was changed twice weekly. At day 14, microglia were detached with Accutase® for 15 min, washed twice in serum free media, or FACS buffer, and processed for downstream applications.

#### Midbrain organoid generation and culture

Neural epithelial stem cells (NESCs; 6,000 cells/well) were seeded into BIOFLOAT™ 96-well plates (faCellitate, F202003), and cultured in maintenance medium consisting of N2B27 supplemented with 3 µM CHIR (Axon CT 99021), 0.2 mM ascorbic acid (Sigma, A4544-100G), 0.5 µM smoothened agonist (SAG, Stemcell Technologies 73412), 2.5 µM SB-431542 (Abcam, ab120163), and 0.1 µM LDN-193189 (Sigma, SML0559) for 2 days.

From day 2 to 4, cells were cultured in patterning medium I (N2B27 supplemented with 0.2 mM Ascorbic Acid, 3 µM CHIR, and 0.5 µM SAG), then in patterning medium II from day 4 to 8 (N2B27 supplemented with 0.2 mM ascorbic acid, 0.7 µM CHIR, and 0.5 µM SAG). From day 8 onward, organoids were cultured in maturation medium of N2B27 supplemented with 0.2 mM ascorbic acid, 10 ng/ml Brain-Derived Neurotrophic Factor (BDNF, Peprotech, 450–02), 10 ng/ml Glial-Derived Neurotrophic Factor (GDNF, Peprotech, 450–10), 1 pg/ml TGF-β3 (Peprotech, 100-36E), 0.5 mM Dibutyryl cyclic-AMP (Biosynth SRO, ND07996), 10 µM DAPT (R&D Systems, 2634/10) and 2.5 ng/ml Activin A (ThermoFisher Scientific, PHC9564). Organoids were maintained under static conditions with biweekly medium changes until day 15 of differentiation.

#### Coculture of midbrain organoids and macrophage precursors (assembloids)

On day 15 of dopaminergic differentiation, 150,000 macrophage precursors were added to each organoid as previously described [17]. Co-culture was maintained in basal microglia differentiation medium supplemented with 100 ng/ml IL-34 (Biolegend, 577906), 10 ng/ml GM-CSF (Biolegend, 572905), 10 ng/ml BDNF, 10 ng/ml GDNF, 10 µM DAPT, and 2.5 ng/ml Activin A. Media were exchanged twice weekly. The duration of co-culture depended on the experimental design (D20 or D75).

### 2D microglia analysis

#### Flow cytometry

For surface marker analysis, microglia were stained in FACS buffer (PBS supplemented with 1% FCS and 5 mM EDTA pH 8.0) with fluorescently conjugated antibodies (Materials, Table S1) for at least 30 min at 4 °C, protected from light. Cells were washed twice, resuspended in FACS buffer and analyzed on a flow cytometer.

For intracellular phosphoprotein detection, cells were fixed in 2% formaldehyde for 10 min, and permeabilized with 0.01% saponin in FACS buffer. Staining and subsequent analysis were performed in 0.01% saponin.

For intracellular cytokine measurement, microglia were stimulated with 100 ng/ml lipopolysaccharide (LPS) for 24 h at D13 of differentiation, with BD GolgiPlug™ (BD Biosciences, 555029) added during the last 6 h of stimulation. Microglia were fixed and permeabilized using the BD Pharmingen™ Transcription Buffer Set (BD Biosciences, 562574) according to the manufacturer’s protocol. This kit was also used to stain intracellular nuclear proteins.

For ROS/mitochondrial analysis, microglia were stained with dichlorofluorescein diacetate (H2-DCF-DA, ThermoFisher Scientific D399) or Mitotracker™ Deep Red and Green (ThermoFisher Scientific, M46753; M46750) for 30 min in non-supplemented DMEM-F12. The cells were washed twice in PBS and analyzed immediately in flow cytometer.

#### Phagocytosis assay

Microglia were incubated with 10 µg/ml Zymosan A (*S.cerevisiae*) BioParticles™ (ThermoFisher Scientific, Z23373) or pHRodo™-Zymosan BioParticles™ Conjugate (ThermoFisher Scientific, P35364) in HBSS medium supplemented with 20mM HEPES and 10% FCS. Cells were incubated for at least 30 min at 37 °C, washed 2 × with PBS, and analyzed by flow cytometry.

#### Glucose uptake assay

Cells were incubated in glucose free DMEM-F12 (non-supplemented) with 50 µM 2-NBDG (ThermoFisher Scientific, N13195) for 1 h at 37°C. Cells were immediately analyzed using a flow cytometer.

#### Metabolic profiling

Microglia (2 × 10^5^ cells/well) were plated in Agilent Seahorse XF DMEM medium (Agilent, 103575–100). The extracellular acidification rate (ECAR) and oxygen consumption rate (OCR) were measured using the XF Glycolytic Stress Test and the XF Cell Mitochondrial Stress Test kits according to the manufacturer’s protocols.

#### Isotopic labelling

Microglia were cultured for 24 h in DMEM-F12 containing [U-^13^C_6_]-glucose (11.1 mmol/L; Cambridge Isotope Laboratories). Metabolite extraction, mass spectrometry, mass isotopomer quantification and determination of fractional carbon contributions were performed as previously described [18], using MetaboliteDetector software package.

#### Cytokine quantification

Proinflammatory cytokines were assessed using Human Inflammatory Cytokine Multiplex ELISA Kit (Arigo Biolaboratories, ARG80929) according to the manufacturer’s protocol. In addition, TNF-α and IL-1β were quantified using human uncoated ELISA Kits from ThermoFisher Scientific (88–7346-77 and 88–7261-77).

#### 2D Immunofluorescence staining

Microglia were differentiated and plated in 96-well glass bottom plate (Cellvis, P96-0-N). The cells were then fixed for 10 min with 4% formaldehyde (Sigma, 100496), and permeabilized using 0.3% Triton X-100 in PBS for 15 min. Microglia were washed three times with PBS and blocked with blocking buffer (3%BSA + 0.3% Triton X-100 in PBS). The cells were stained overnight at 4 °C with primary antibodies diluted in blocking buffer. The cells were rinsed three times and incubated with secondary antibodies in blocking buffer for 2 h at room temperature. After two PBS washes, the samples were resuspended in PBS + 0.1% Na-Azide and imaged using Zeiss LSM710 Confocal Laser Scanning Microscope.

#### RNA extraction and RT-PCR

RNA isolation was done using RNeasy Mini Kit (Qiagen, 74104) according to the manufacturer’s protocol. cDNA was prepared using High-Capacity RNA-to-cDNA™ Kit (ThermoFisher Scientific, 4387406). RT-PCR was performed using Green-Taq polymerase with 50ng of cDNA per reaction. The primers used are listed under ‘Oligonucleotides’. The reactions were run on an ABI 7500 HT Fast qRT-PCR instrument. Data were normalized to control gene and analyzed using ΔΔCt method as previously described.

#### RNA sequencing

Libraries were prepared using Novogene’s property library prep protocols (Novogene NGS RNA Library Prep Set PT042 for 250–300 bp insert cDNA library). The indices were incorporated to multiplex multiple samples. mRNA was purified using poly-T oligo-attached magnetic beads. After fragmentation, the first strand cDNA was synthesized using random hexamer primers, followed by a second strand cDNA synthesis. Library preparation was finalized following end repair, A-tailing, adapter ligation, and size selection. After amplification and purification, the insert size of the library was validated on an Agilent 2100 and quantified using RT-PCR. Libraries were sequenced using Illumina NovaSeq 6000 S4 flow cell with PE150.

#### Transcriptomic analysis

The data were analyzed and visualized with RStudio (R version 4.3.3). Raw sequencing reads were processed using the Rsubread package (version 2.10.4) for alignment and quantification. Paired-end reads were aligned to the human reference genome GRCh38 using the Subread aligner with default parameters. The alignment quality showed high mapping rates with over 98% of reads successfully mapped across all samples. Gene-level counts were generated using the featureCounts function from Rsubread. Differential gene expression analysis was done using the DESeq2 pipeline [19]. GeneGO Metacore was used for gene set enrichment analysis (https://clarivate.com; https://portal.genego.com). The GEO accession number for the RNA-seq data from the healthy and mutant microglia in this study is GSE282494.

#### Metabolic modelling

Context-specific metabolic models were reconstructed by integration of RNA sequencing data into the Recon 3 generic human metabolic model [20] using rFASTCORMICS pipeline [21]. With this workflow, transcripts per million (TPM) normalized gene expression was used as a determinant of the presence of metabolic reactions models. In addition, metabolic networks were constrained by the composition of cell culture media, defying the availability of metabolites which can be taken up by cells. A separate metabolic model was generated for each LRRK2-WT and LRRK2-G2019S microglia. Models were generated and analyzed in MATLAB (MathWorks, v.2019b) using the COBRA toolbox [22]. Using the setdiff function in MATLAB shared reactions across the WT and MUT models were identified. Unique reactions for each condition (only present in the WT or MUT condition) were determined by the intersect function in MATLAB and assigned to the corresponding subsystem of Recon 3. Subsystems with five or more unique reactions, representing at least 10% of the total reaction number in the respective subsystem in Recon 3 for WT or MUT conditions, were selected for further investigation.

### 3D assembloid analysis

#### Flow cytometry

The assembloids were incubated for 30 min at 37 °C in 100 µl Accutase® under an orbital shaker. The assembloid underwent mechanical dissociation into a single cell suspension with a 1000 µl pipette followed by a 200 µl pipette. The cell suspensions were washed twice with assay media or PBS. The assembloids were fixed and permeabilized with a BD Pharmingen™ Transcription Buffer Set. The cells were stained for neuronal markers: dopaminergic neurons (TH), neurons (MAP2). The samples were analyzed using flow cytometer BD LSR Fortessa ™.

#### Immunofluorescence staining

The assembloids were fixed overnight in 4% paraformaldehyde at RT and washed 3 × with PBS for 15 min. The assembloids were then embedded in 3% low melting point agarose (Biozym Scientific GmbH, 840100) and cut into 70 µm sections using Leica VT1000s vibratome. One assembloid was sliced into approximately 10–15 sections depending on the size. The sections were permeabilized in 0.5% Triton X-100 in PBS for 2 h. The samples were blocked with a mixture of 2.5% BSA and 2.5% donkey serum in PBS for 2 h at RT. The sections were then incubated for 48 h at 4 °C with primary antibodies diluted in blocking buffer. The sections were washed 3 × for 10 min with 0.01% Triton X-100 in PBS and incubated with secondary antibody for 2 h diluted in blocking buffer. The list of the primary and secondary antibodies is listed in Materials. The sections were washed 3 × for 10 min with 0.01% Triton X-100 in PBS, followed by one wash with H_2_O. Finally, the sections were mounted on DBM Teflon® slides (De Beer Medicals, BM-9244) using Fluoromount-G® mounting media (Southern Biotech, 0100–01).

#### Image analysis

Image acquisition and analysis was done using the established pipeline in the group [23]. All sections of the assembloid (x, y and z fields) were acquired with a 20 × objective using Yokogawa CV8000 standalone high content screening confocal microscope and the Cell Voyager coupled with Wako software. Image quantification was done using in-housed MATLAB scripts, from two sections per organoid per cell line and from at least three independent assembloid derivations.

#### Data analysis and statistics

The data were analyzed using GraphPad Prism 10.1.2. A normality test was performed using the Shapiro test. If not stated otherwise, outlier removal was performed using the ROUT method Q 1% in GraphPad. For nonnormally distributed data, two-sided Wilcoxon test or Kruskal–Wallis with Dunn’s multiple comparison test and Benjamini–Hochberg correction was implemented. For normally distributed data, Welch’s t-test or one-way ANOVA with Tukey’s multiple comparison test were performed. Significant P values are represented with asterisks in the order *P* < 0.05 *, *P* < 0.01 **, *P* < 0.001 ***, *P* < 0.0001 ****. The error bars represent mean ± SD.

## Results

### LRRK2-G2019S mutation does not affect iPSC-derived microglial differentiation and identity

**Fig. 1:**
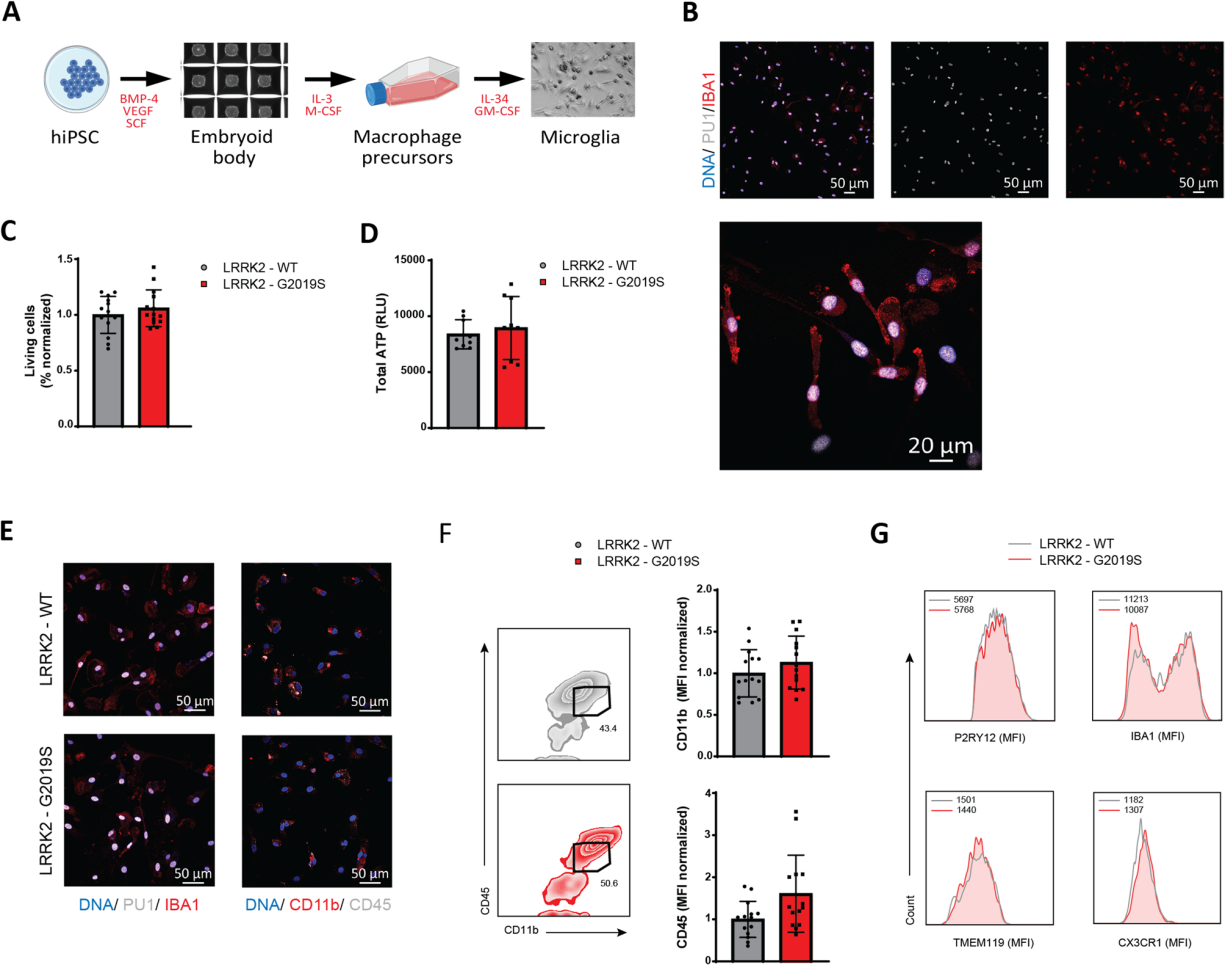
Generation and characterization of iPSC-derived microglia identity show no alteration in microglia harboring the LRRK2-G2019S mutation. **A** Schematic representation of the generation of iPSC-derived microglia. **B** Representative immunostaining image of healthy iPSC-derived microglia with Hoechst, PU1, and IBA1. **C** Flow cytometry analysis of LRRK2-WT and LRRK2-G2019S microglia viability upon differentiation using the Zombie NIR™ Fixable viability kit. Data are shown as mean ± SD (*n* = 3–4), pooled from three independent trials **D** Luminescence-based quantification of cellular ATP levels in LRRK2-WT and LRRK2-G2019S microglia. Data are shown mean ± SD (*n* = 3–4), pooled from three independent trials. **E** Representative immunostaining images of LRRK2-WT and LRRK2-G2019S microglia with Hoechst, PU1, IBA1 (left) and Hoechst, CD11b, CD45 (right). **F** Flow cytometry analysis of mature microglia (CD11b^hi^CD45^int^) (left) and representative analysis of mean fluorescence intensity of CD11b and CD45 (right) in LRRK2-WT and LRRK2-G2019S microglia. Data are shown as mean ± SD (*n* = 3–4), pooled from three independent trials. **G**. Representative analysis of mean fluorescence intensity of microglia identity markers (P2RY12, IBA1, TMEM119, CX3CR1) in mature LRRK2-WT vs LRRK2-G2019S microglia using flow cytometry

In this study, we used iPSCs from four healthy individuals and four PD patients harboring the LRRK2-G2019S mutation (Table 1). Initially, embryoid bodies were formed from the iPSCs and cultured in a flask to produce macrophage precursors (Fig. 1A). These precursors were harvested and differentiated for 14 days to generate mature microglia, according to the previously established protocol [17, 24]. Healthy iPSC derived microglia expressed typical macrophage and microglial markers such as IBA1 and PU1 (Fig. 1B). After validating our robust microglial generation protocol, we compared microglia from healthy individuals and those with the LRRK-G2019S mutation.

Despite reports of LRRK2-related toxicity resulting in 10–70% cell death, other studies found no effect on cell viability [25]. In our system, we observed no differences in cell viability as assessed by flow cytometry (Fig. 1C), or total ATP levels using CellTiter-GLO® luminescence assay (Fig. 1D). Additionally, the presence of the LRRK2-G2019S mutation did not impact embryoid body formation or the yield of macrophage precursors (Figure S1A). Furthermore, morphological assessments via flow cytometry (FSC/SSC) revealed no differences in cell size or complexity between healthy and mutant microglia (Figure S1B).

Upon stimulation IL-34 and GM-CSF, macrophage precursors mature into microglial cells [24]. Mature microglia express markers, including general macrophage markers (CD11b, CD45, IBA1, PU1) as well as specific microglial markers (P2RY12, CX3CR1, and TMEM119) [26]. Both wild-type (WT) and LRRK2-G2019S microglia showed comparable expressions of these markers, as confirmed by immunofluorescence and flow cytometry (Fig. 1E-G*, *S1C). Together, these data indicate that the LRRK2-G2019S mutation does not alter the differentiation efficiency, identity, or basic morphology of iPSC-derived microglia.

### LRRK2-G2019S microglia exhibit heightened immune activation and inflammatory phenotypes

Despite normal differentiation, LRRK2-G2019S microglia exhibit increased immune activation and proinflammatory properties. Previous studies have implicated LRRK2 polymorphisms in autoinflammatory diseases including inflammatory bowel disease (IBD), Crohn’s disease, and tuberculosis, indicating a significant connection to immune functions [27]. Notably, increased expression of LRRK2 has been observed in immune cells in response to proinflammatory signals [28]. We therefore sought to determine the impact of LRRK2-G2019S mutation on microglial inflammatory functions in iPSC-derived microglia.

**Fig. 2:**
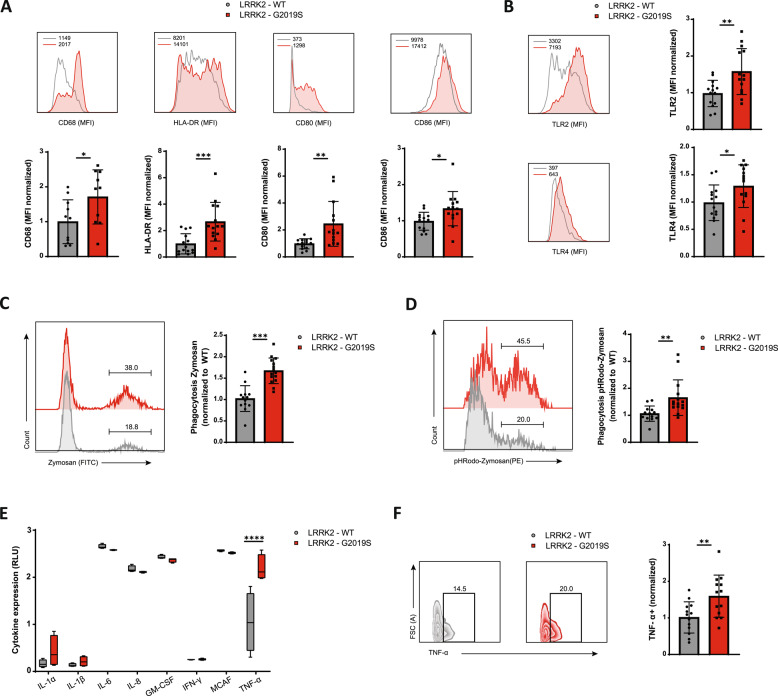
Immunological phenotyping of LRRK2-G2019S microglia showed heightened activation and inflammatory profiles. **A** Representative analysis of mean fluorescence intensity (top) and the quantification (bottom) of activation markers (CD68, HLA-DR, CD80, and CD86) of LRRK2-WT and LRRK2-G2019S microglia. Data are shown as mean ± SD (*n* = 3–4), pooled from four independent trials. **B** Representative analysis of mean fluorescence intensity (left) and the quantification (right) of pathogen recognition receptors TLR2 and TLR4 in LRRK2-WT and LRRK2-G2019S microglia. Data are shown as mean ± SD (*n* = 3–4), pooled from four independent trials. **C** Flow cytometry analysis of microglia phagocytosis in LRRK2-WT and LRRK2-G2019S microglia using fluorescently labelled Zymosan beads. Representative images are shown on the left, quantification on the right. Data are shown as mean ± SD (*n* = 3–4), pooled from four independent trials. **D** Flow cytometry analysis of microglia phagocytosis in LRRK2-WT and LRRK2-G2019S microglia using pH-sensitive fluorescent Zymosan beads. Representative images are shown on the left, quantification on the right. Data are shown as mean ± SD (*n* = 3–4), pooled from four independent trials. **E** Enzyme-linked immunosorbent assay of human inflammatory cytokines in LRRK2-WT and LRRK2-G2019S microglia using the Human Inflammatory Cytokine Multiplex ELISA Kit from Arigo Biolaboratories. Data are shown as mean ± SD (*n* = 4), representative of two independent trials. **F** Flow cytometry analysis of intracellular TNF-α in LRRK2-WT and LRRK2-G2019S microglia upon 24 h stimulation with LPS. Representative images are shown on the left, quantification on the right. Data are shown as mean ± SD (*n* = 3–4), pooled from four independent trials. *p < 0.05, **p < 0.01, ***p < 0.001, ****p < 0.0001

Flow cytometry analysis showed that CD68, a lysosomal protein indicative of phagocytic activity, was significantly upregulated in LRRK2-G2019S microglia compared to healthy controls (Fig. 2A). Markers associated with antigen presentation and costimulation, including HLA-DR, CD80 and CD86, were elevated in mutant microglia relative to that in control microglia (Fig. 2A). However, other activation markers such as CD69, CCR6, and the exhaustion marker PD-1 were unchanged (Figure S2A).

One of the key functions of microglia is the uptake of antigens for removal or presentation. Microglia possess several pathogen recognition receptors, namely Toll-like receptors (TLRs). TLR signaling and phagocytosis are hallmarks of the microglia-mediated immune response to infections [29]. The activation of TLR2 has been associated with neuronal injury [30]. Our data revealed that LRRK2-G2019S microglia presented increased levels of both TLR2 and TLR4, suggesting a potential increase in phagocytic activity (Fig. 2B). To assess microglial phagocytosis, we used fluorescence labelled Zymosan, a TLR2 agonist derived from yeast. Following incubation and thorough washing to remove unbound particles, LRRK2-G2019S microglia showed significantly greater uptake of zymosan than healthy microglia did (Fig. 2C). Using a more advanced fluorescence Zymosan particle coupled with a pH-sensitive pHRodo, which fluoresces only when the particle is digested in the lysosome, we confirmed an increased phagocytic rate in LRRK2-G2019S microglia (Fig. 2D).

Another critical function of microglia is the production of cytokines. In response to environmental cues, microglia can produce a range of pro- and anti-inflammatory cytokines. Proinflammatory cytokines such as IL-1, IL-6, IFN, and TNF-α have been implicated in the pathogenesis of PD [31]. Our results indicated that various inflammatory cytokines were similarly expressed between healthy and LRRK2-G2019S microglia. However, upon LPS stimulation, LRRK2-G2019S microglia uniquely secreted higher levels of TNF-α into the culture media than the control microglia (Fig. 2E). This finding was corroborated by flow cytometry, which revealed significant upregulation of intracellular TNF-α in LRRK2-G2019S microglia (Fig. 2F*, *Figure S2B). Interestingly, other inflammatory cytokines were expressed at similar levels in both groups (Fig. 2E, Figure S2B). In summary, our data suggest that the LRRK2-G2019S mutation enhances the inflammatory activity and function of microglia, which is primarily mediated by increased TNF-α expression.

### Transcriptomic profiling reveals altered inflammatory profiles and ROS metabolism in LRRK2-G2019S microglia

**Fig. 3:**
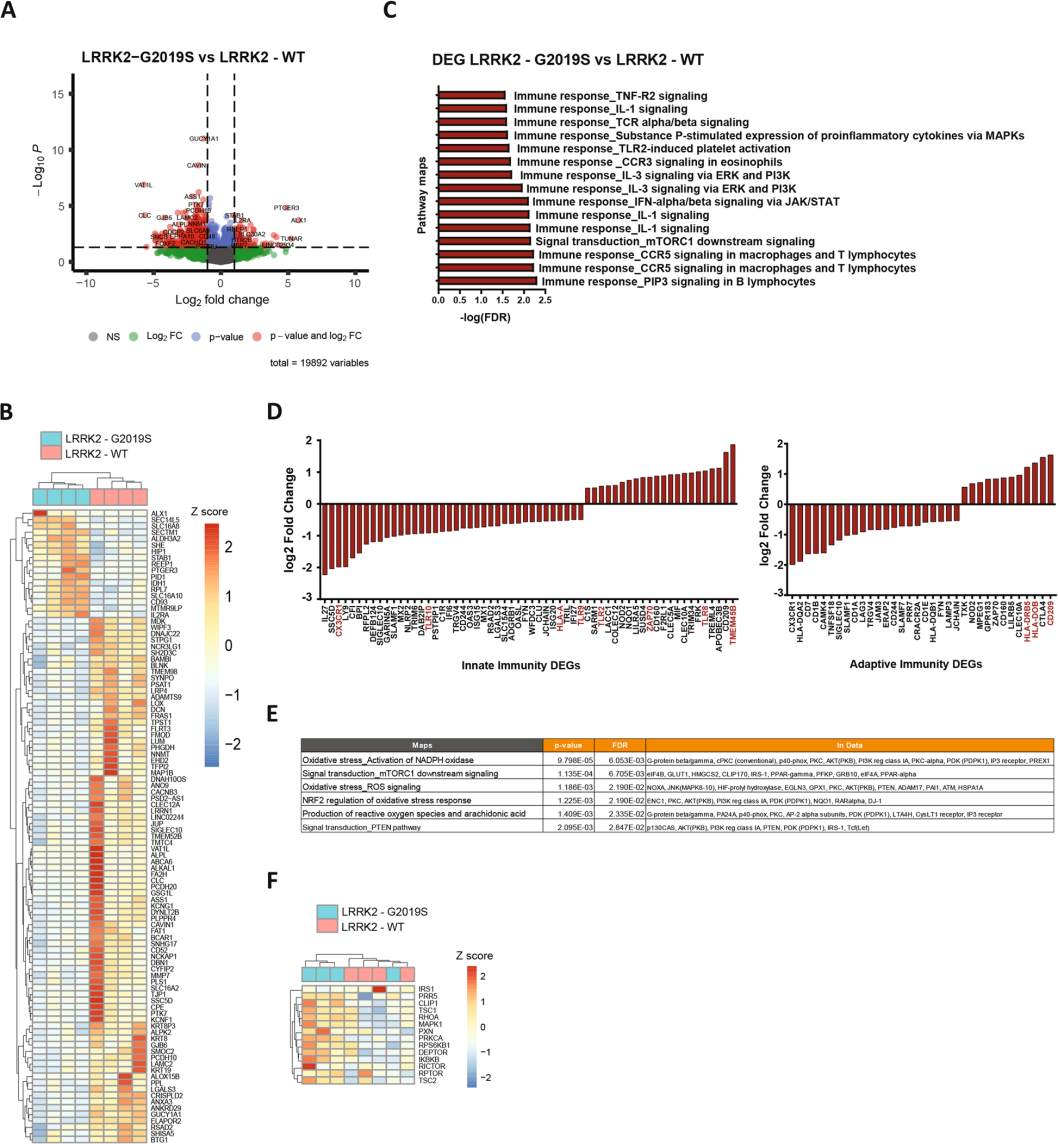
Transcriptomic profiling of LRRK2-G2019S microglia revealed more pro-inflammatory microglia phenotypes. **A** Volcano plot showing all genes with genes having log_2_FC > 1 and *p*-value < 0.05 highlighted, indicating differentially expressed genes between LRRK2-WT and LRRK2-G2019S microglia. **B** Top 50 differentially expressed genes (DEGs) between LRRK2-WT and LRRK2-G2019S microglia with the lowest *p* value. **C** Enrichment analysis using Metacore, showing top pathway maps involved in immunity. **D** Fold changes of genes from the GO term of innate and adaptive immunity with log_2_FC > 0.5. **E** Metacore enrichment analysis of pathways associated with cellular metabolism. **F** Heatmap of mTOR-regulated genes distinguishing LRRK2-WT from LRRK2-G2019S microglia upon hierarchical clustering

To gain a deeper understanding of the molecular changes induced by the LRRK2-G2019S mutation in microglia, we performed a bulk RNA sequencing analysis. We included four healthy controls and four iPSC-derived microglia carrying the LRRK2-G2019S mutation. The transcriptomic data identified over 970 genes whose expression significantly differed between control and mutant microglia (Fig. 3A, B). Pathway analysis revealed that several inflammatory signaling pathways were differentially regulated in LRRK2-G2019S microglia compared with healthy controls. Among these, TNF and IL-1 signaling, both of which are critical mediators of immune responses and inflammation, were notably altered (Fig. 3C).

Our transcriptomic analysis also revealed distinct alterations in genes related to microglial inflammatory processes such as phagocytosis and inflammation (Fig. 3C). Genes involved in pathogen recognition and phagocytosis including TLR2, TLR4, TLR8, TLR9, and TLR10 – exhibited altered expression in LRRK2-G2019S microglia. Similarly, the expression of ZAP70, a key molecule involved in immune synapse formation, was altered (Fig. 3D*, *Figure S3A). Additionally, genes involved in antigen presentation, such as HLA-DRB5, HLA-DOB, and CD209 (DC-SIGN) also displayed increased expression in mutant microglia (Fig. 3D, Figure S3A). These findings highlight the broad impact of the LRRK2-G2019S mutation on microglial profiles.

Given the observed upregulation of inflammatory markers at both the RNA and protein levels in LRRK2-G2019S microglia, we further investigated the underlying cellular mechanism contributing to this heightened immune response. Recent studies have emphasized the pivotal role of immune metabolism in regulating cell development, proliferation, and function. Immune cells dynamically rewire their metabolic preferences in response to environmental cues and functional demands. We hypothesized that the LRRK2-G2019S mutation might impair microglial metabolism, contributing to their abnormal immune function.

Enrichment analysis identified several altered pathways in LRRK2-G2019S microglia, many of which were related to metabolism *(Supplemental information* Table 1). Notably, pathways such as reactive oxygen species (ROS) metabolism and mammalian target of rapamycin complex 1 (mTORC1) signaling were enriched in mutant microglia (Fig. 3E). mTORC1, which plays a key role in regulating cellular metabolism and immune responses, has been implicated in microglial functions [32, 33]. Similarly, our data revealed that genes regulating mTORC1 activity, including TSC1, TSC2, and RPTOR, were differentially expressed in LRRK2-G2019S microglia (Fig. 3F).

To gain a more comprehensive view of the metabolic changes associated with the LRRK2-G2019S mutation, we constructed context-specific metabolic models by integrating the RNA sequencing data with the Recon3 generic human metabolic model, using the rFASTCORMICS pipeline [20]. This model predicted significant alterations in several metabolic pathways, including energy metabolism, sugar metabolism, amino acid metabolism, and lipid metabolism. Importantly, the mutant microglia exhibited unique metabolic reactions that were absent in the healthy controls (Figure S3B). Further analysis identified the most dysregulated metabolic subsystems in LRRK2-G2019S microglia. These included ROS metabolism (particularly glutathione metabolism), lipid metabolism and glycolysis, all of which play key roles in maintaining cellular homeostasis and responding to stress (Figure S3C). Dysregulation of these metabolic pathways is likely to contribute to the pathological phenotypes observed in LRRK2-G2019S microglia.

### LRRK2-G2019S microglia upregulate glycolysis and downregulate glucose-derived serine synthesis

**Fig. 4:**
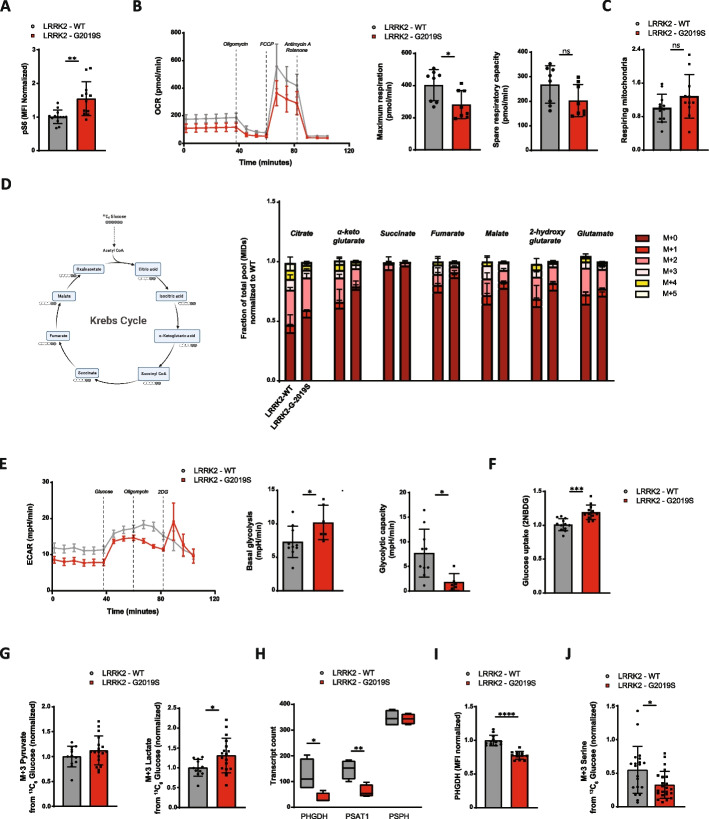
Metabolic analysis indicated metabolic reprogramming in LRRK2-G2019S microglia. **A** Flow cytometry analysis of protein S6 phosphorylation in LRRK2-WT and LRRK2-G2019S microglia. Data are shown as mean ± SD (*n* = 3–4), pooled from four independent trials. **B** Measurement of oxygen consumption rate (OCR) in LRRK2-WT and LRRK2-G2019S microglia using the Seahorse Cell Mitochondrial Stress Test. Maximum respiration and spare respiratory capacity were quantified (right). Data are shown as mean ± SD (*n* = 3–4), representative from three independent trials. **C** Flow cytometry analysis of respiring mitochondria, calculated by the ratio between Mitotracker Deep Red™ (marking active mitochondria) and Mitotracker Green ™ (marking total mitochondria). Data are shown as mean ± SD (*n* = 3–4), pooled from four independent trials. **D** Flux map of carbon metabolism (left) and mass isotopomer distribution of TCA cycle metabolites in LRRK2-WT and LRRK2-G2019S microglia incubated with U-^13^C_6_-glucose during the final 24 h of differentiation. Data are shown as mean ± SD (*n* = 3–4), pooled from three independent trials. **E** Measurement of extracellular acidification rate (ECAR) in LRRK2-WT and LRRK2-G2019S microglia using Seahorse Glycolysis Stress Test. Basal glycolysis and glycolytic capacity were quantified (right). Data are shown as mean ± SD (*n* = 3–4), representative from three independent trials. **F** Flow cytometry analysis of glucose uptake rate in LRRK2-WT and LRRK2-G2019S microglia using fluorescent glucose 2-NBDG. Data are shown as mean ± SD (*n* = 3–4), pooled from four independent trials. **G** Mass isotopomer distribution of pyruvate (M + 3) and lactate (M + 3) in LRRK2-WT and LRRK2-G2019S microglia incubated with U-^13^C_6_-glucose during the final 24 h of differentiation. Data are shown as mean ± SD (*n* = 3–4), pooled from three independent trials. **H** Transcript count of serine synthesis genes PHGDH, PSAT1, and PSPH in LRRK2-WT and LRRK2-G2019S microglia. **I** Flow cytometry analysis of intracellular protein PHGDH in LRRK2-WT and LRRK2-G2019S microglia. Data are shown as mean ± SD (*n* = 3–4), pooled from four independent trials. **J** Mass isotopomer distribution of serine synthesis (M + 3) in LRRK2-WT and LRRK2-G2019S microglia incubated with U-.^13^C_6_-glucose during the final 24 h of differentiation. Data are shown as mean ± SD (*n* = 3–4), pooled from three independent trials. *p < 0.05, **p < 0.01, ***p < 0.001, ****p < 0.0001

In our previous analysis, transcriptomic profiling and metabolic modelling showed changes in microglia harboring the LRRK2-G2019S mutation. mTOR plays a crucial role in integrating extracellular and intracellular signals to regulate cellular metabolism and growth [34]. Given the observed changes in metabolic homeostasis at the transcriptomic level, we next sought to determine the functional status of mTOR by measuring its phosphorylation using flow cytometry. Interestingly, the phosphorylation of mTOR was comparable in LRRK2-G2019S microglia (Figure S4A). However, we detected a significant increase in the expression of phosphorylated S6 (pS6), a key downstream target of mTORC1 in LRRK2-G2019S microglia compared with that in healthy controls (Fig. 4A).

Since mTORC1 signaling is known to regulate major metabolic pathways, such as mitochondrial respiration and glycolysis, the upregulation of mTORC1 in LRRK2-G2019S microglia could impact these processes. To assess mitochondrial function, we performed a mitochondrial stress test using Seahorse flux analysis. Although the spare respiratory capacity remained similar, the maximum respiration rate as well as proton leak was significantly lower in LRRK2-G2019S microglia (Fig. 4B, SF4B).

Interestingly, the ratio of mitochondria with intact membrane potential (stained with MitoTracker DeepRed ™) to total mitochondrial mass (stained with MitoTracker Green™) was comparable between the mutant and control microglia, suggesting that mitochondrial quantity and membrane integrity were not compromised (Fig. 4C). Furthermore, the level of Ki-67 staining, a marker for cell proliferation, showed no differences between LRRK2-G2019S and healthy microglia (Figure S4C). Similarly, total ROS levels, as assessed using H2-DCFDA probe (Figure S4D), as well as mitochondria ROS (data not shown), were also comparable between the two groups. Normal ROS levels suggest that LRRK2-G2019S microglia do not experience excessive oxidative stress, despite their heightened metabolism and immune activity. These results indicate that mitochondrial function is largely preserved in LRRK2-G2019S microglia, and that the reduced reliance on mitochondrial respiration suggests a shift toward alternative energy pathways to sustain microglial activity.

To further explore the altered metabolic flux in LRRK2-G2019S microglia, we performed metabolic tracing using uniformly labelled ^13^C_6_ glucose. This approach allowed us to track the fate of glucose through various metabolic pathways. Pyruvate, the primary end product of glycolysis, can be used in the mitochondria to fuel the tricarboxylic acid (TCA) cycle. The mass isotopologue distribution (MID) showed no significant differences in the levels of TCA cycle intermediates – such as citrate, α-ketoglutarate, succinate, fumarate, and malate – between healthy and LRRK2-G2019S microglia (Fig. 4D). These findings align with the mitochondrial respiration results, further highlighting that the LRRK2-G2019S mutation does not significantly affect mitochondrial metabolism in microglia.

In resting immune cells, including microglia, energy production is typically reliant on mitochondrial metabolism; however, upon activation, these cells rewire their metabolism into glycolysis, a faster but less efficient metabolic pathway, to meet the increased energy demands [8]. Given the elevated immune activation in LRRK2-G2019S microglia, we suspected that glycolysis might be upregulated in these cells. To test this hypothesis, we performed a glycolytic stress test using Seahorse flux analysis. Indeed, basal glycolysis was elevated in LRRK2-G2019S microglia compared with healthy controls (Fig. 4E). However, their glycolytic capacity, defined as the maximum rate of glycolysis under stress conditions, was significantly reduced (Fig. 4E).

We further confirmed this metabolic shift by assessing glucose uptake using 2-NBDG, a fluorescent glucose analog. Compared with healthy microglia, LRRK2-G2019S microglia exhibited significantly greater glucose uptake (Fig. 4F). Instead of entering the TCA cycle, a significant portion of pyruvate generated from glycolysis was converted into lactate, as indicated by increased levels of M + 3 glucose-derived lactate (Fig. 4G). These data suggest that LRRK2-G2019S microglia undergo metabolic reprogramming and rely more on glycolysis to support their hyperactive immune function.

Glycolysis not only generates pyruvate but also produces intermediate metabolites that serve as precursors for other biosynthetic pathways. Specifically, 3-phosphoglycerate serves as a substrate for serine biosynthesis. Interestingly, two key genes involved in serine biosynthesis – PHGDH and PSAT1 – were significantly downregulated in LRRK2-G2019S microglia (Fig. 4H). PHGDH catalyzes the first and rate-limiting steps of serine synthesis. Flow cytometry confirmed a significant reduction in PHGDH expression in LRRK2-G2019S microglia compared with that in control microglia (Fig. 4I).

To further validate these findings, we traced the incorporation of glucose into serine using ^13^C_6_ glucose. The results revealed significantly lower levels of M + 3 glucose-derived serine in LRRK2-G2019S microglia than in control microglia, indicating an impaired serine biosynthetic pathway (Fig. 4J). The reduction in serine production may reflect a compensatory shift to drive enhanced glycolysis due to the LRRK2-G2019S mutation, which needs to be further elaborated. In summary, our data show that LRKR2-G2019S microglia exhibit metabolic reprogramming, characterized by elevated glycolysis and impaired serine synthesis, which might be targeted to restore normal microglial functions in PD.

### LRRK2-G2019S microglia induce degeneration of dopaminergic neurons in midbrain organoids

Neuroinflammation has been implicated in the progressive degeneration of dopaminergic neurons in PD [35, 36]. Here, we demonstrated that LRRK2-G2019S microglia upregulate immune activity, which is characterized by increased phagocytosis and elevated production of the proinflammatory cytokine TNF-α. Given the established role of neuroinflammation in neuronal injury, we sought to explore how microglial immune activation contributes to dopaminergic neuron loss, with a specific focus on the pathological effects of the LRRK2-G2019S mutation.

**Fig. 5:**
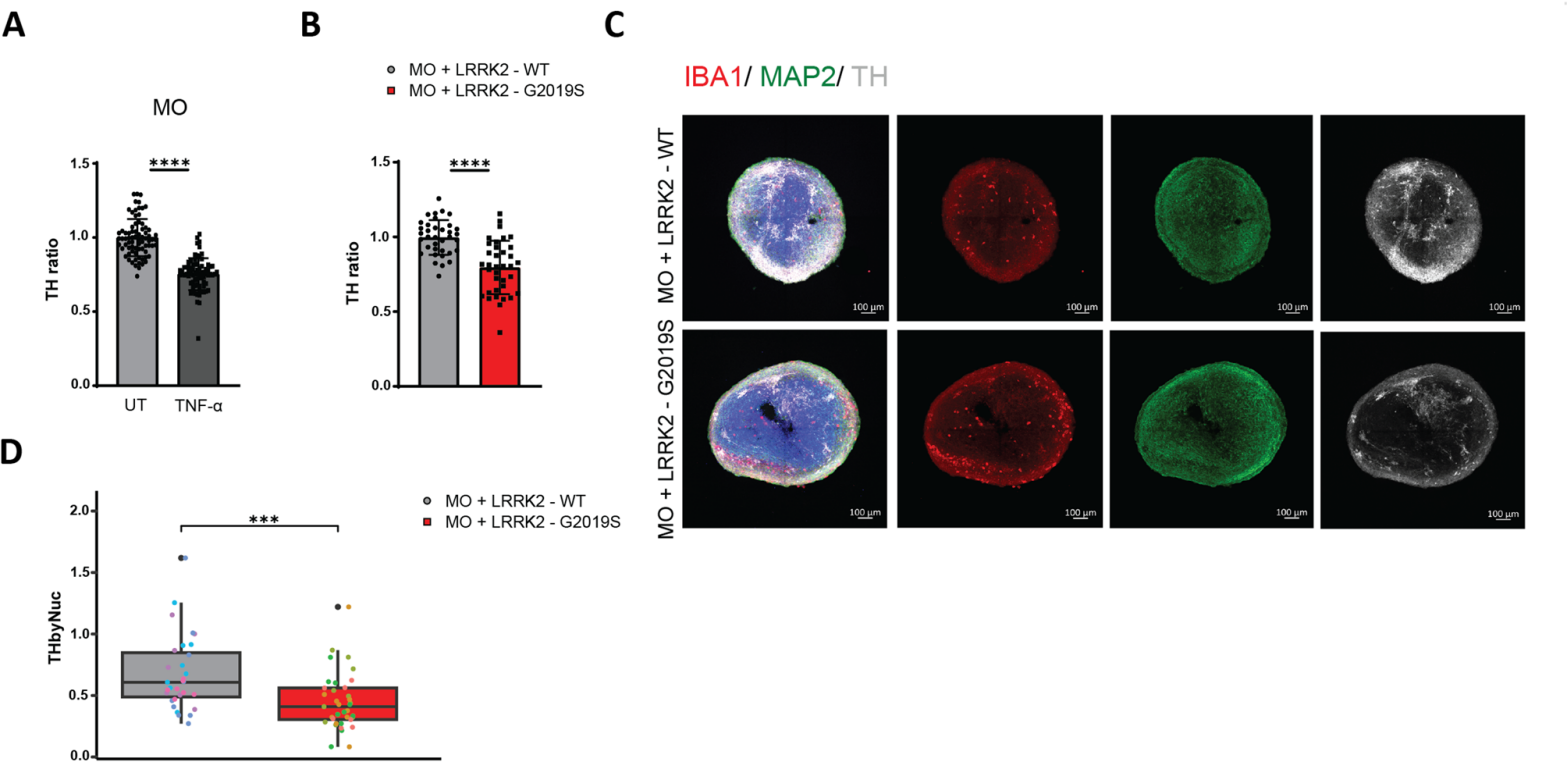
LRRK2-G2019S microglia induced dopaminergic neuron degeneration in midbrain organoids. **A** The number of dopaminergic neurons, represented by the total TH + cells over total MAP2 + neurons, in midbrain organoids treated with or without 100 pg/ml TNF-α. Data are shown as mean ± SD (*n* = 3), pooled from three independent trials. **B** The number of dopaminergic neurons in midbrain organoids co-cultured with LRRK2-WT and LRRK2-G2019S microglia. Data are shown as mean ± SD (*N* = 3, *n* = 4), pooled from three independent trials. **C** Representative confocal image of a 70 μm midbrain organoids section with LRRK2-WT (top) or LRRK2-G2019S (bottom) microglia, immunostained with Hoechst, TH, MAP2, and IBA1. **D** High-content automated image analysis of immunofluorescence staining of dopaminergic neurons in assembloids (shown in C), expressed as the proportion of TH + cells normalized by total nuclei. Dotted color showed individual midbrain organoids. Data are shown as mean ± SD (*N* = 3, *n* = 4), pooled from three independent trials. *p* < 0.05, **p < 0.01, ***p < 0.001, ****p < 0.0001

To do so, we used midbrain organoids derived from healthy individual iPSCs [23]. To simulate the inflammatory conditions associated with LRRK2-G2019S microglia, we treated these healthy midbrain organoids with exogenous TNF-α. This cytokine exposure led to a significant reduction in the number of dopaminergic neurons, by up to 30% compared with that in untreated controls (Fig. 5A). Interestingly, in the co-culture of organoids with LRRK2-G2019S microglia, TNF-α neutralizing antibody rescued dopaminergic neuron degeneration to the WT level (*SF4E*). This result suggests that inflammatory signaling alone is sufficient to induce neuronal injury, mimicking aspects of LRRK2-G2019S microglial inflammation in a TNF-α-dependent manner and that blocking TNF-α protect dopaminergic neurons.

To further capture the complexity of the brain’s microenvironment and the contribution of microglia to dopaminergic neuron loss, we integrated iPSC-derived microglia into the midbrain organoids [17]. This coculture approach allowed us to assess the direct effects of both healthy and LRRK2-G2019S mutant microglia on neuronal health and survival. Flow cytometry analysis showed that healthy midbrain organoids cocultured with LRRK2-G2019S microglia had significantly lower levels of tyrosine hydroxylase (TH) positive cells, the rate-limiting enzyme in dopamine synthesis and a surrogate marker of dopaminergic neurons. In contrast, organoids cocultured with healthy control microglia retained higher levels of TH, suggesting that the mutant microglia had a neurotoxic effect on dopaminergic neurons (Fig. 5B).

This trend was further confirmed by imaging analysis. Immunostaining of the organoids revealed that the incorporation of both healthy and LRRK2-G2019S microglia was comparable. The total number of MAP2 neurons in both organoids were comparable. In contrast, the number of TH positive dopaminergic neurons was significantly reduced in LRRK2-G2019S microglia containing midbrain organoids (Fig. 5C, D). These data demonstrate that neuroinflammation driven by LRRK2-G2019S microglia is associated with reduced dopaminergic neuron markers in co-cultured midbrain organoids. Elevated levels of TNF-α produced by the mutant microglia appear to play a central role in this neurotoxicity.

### Oxamic acid reduces immune activity and attenuates dopaminergic neuron loss

The finding that midbrain organoids cocultured with LRRK2-G2019S microglia experienced significant dopaminergic neuronal loss (Fig. 5B, C), indicates that microglia-mediated neuroinflammation plays a central role in PD pathogenesis. These results underscore the potential of targeting microglia as a strategy for disease modification. Given that impaired serine biosynthesis results in increased levels of mTOR and glycolysis, we speculated that targeting the metabolic dysregulation could mitigate neuroinflammation and prevent dopaminergic neuron loss.

To address this hypothesis, we aimed to target the serine-mTOR-glycolysis axis. We used oxamic acid, a pyruvate analog that inhibits lactate dehydrogenase by forming an inactive complex with the enzyme [37, 38]. Oxamic acid-mediated inhibition of lactate dehydrogenase has been shown to reduce glycolysis and mTOR-dependent metabolic reprogramming in cancer cells [39, 40]. Here, we aimed to determine whether targeting glycolysis in LRRK2-G2019S microglia would alleviate their inflammatory toxicity and rescue TH neuronal levels.

**Fig. 6:**
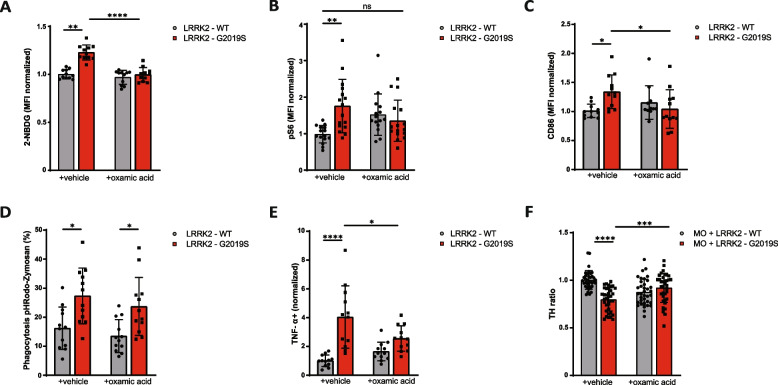
Targeting microglia metabolism with oxamic acid rescued LRRK2-G2019S microglia overt activation and preserved dopaminergic neurons in midbrain organoids. **A** Flow cytometry analysis glucose uptake rate using fluorescent glucose 2-NBDG in LRRK2-WT and LRRK2-G2019S microglia treated with or without oxamic acid. Data are shown as mean ± SD (*n* = 3–4), pooled from three independent trials. **B** Flow cytometry analysis of protein S6 phosphorylation in LRRK2-WT and LRRK2-G2019S microglia treated with or without oxamic acid. Data are shown as mean ± SD (*n* = 3–4), pooled from three independent trials. **C** Flow cytometry analysis of CD86 in LRRK2-WT and LRRK2-G2019S microglia treated with or without oxamic acid. Data are shown as mean ± SD (*n* = 3–4), pooled from three independent trials. **D** Flow cytometry analysis of microglia phagocytosis using pH-sensitive fluorescently labelled Zymosan beads in LRRK2-WT and LRRK2-G2019S microglia treated with or without oxamic acid. Data are shown as mean ± SD (*n* = 3–4), pooled from three independent trials. **E** Flow cytometry analysis of intracellular TNF-α in LRRK2-WT and LRRK2-G2019S microglia treated with or without oxamic acid. Data are shown as mean ± SD (*n* = 3–4), pooled from three independent trials. **F** Number of dopaminergic neurons in midbrain organoids co-cultured with LRRK2-WT and LRRK2-G2019S microglia treated with or without oxamic acid. Data are shown as mean ± SD (*N* = 3, *n* = 4), pooled from three independent trials

As predicted, compared with no treatment, oxamic acid significantly reduced glucose uptake in LRRK2-G2019S mutant microglia (Fig. 6A). This effect was selective, as glucose uptake in healthy microglia remained unaffected by the treatment, suggesting that oxamic acid specifically targets hyperactive glycolysis in LRRK2-G2019S microglia. Additionally, the elevated levels of pS6 in LRRK2-G2019S microglia were normalized to the control level upon treatment with oxamic acid (Fig. 6B).

Next, we examined whether oxamic acid attenuate LRRK2-G2019S microglia immune hyperactivity. Flow cytometry analysis showed a significant reduction in the expression of the costimulatory molecules CD86 in LRRK2-G2019S microglia following oxamic acid treatment (Fig. 6C). Interestingly, while the inflammatory markers were suppressed, oxamic acid treatment did not affect the phagocytic capacity of LRRK2-G2019S microglia (Fig. 6D). However, the levels of the inflammatory cytokine TNF-α were significantly reduced in LRRK2-G2019S microglia than in the untreated microglia (Fig. 6E). These findings confirm that targeting microglial metabolism can alter the course of immune activation, possibly mitigating the toxic effect of LRRK2-G2019S microglia on dopaminergic neurons.

Finally, to determine whether the rescue of metabolic and inflammatory phenotypes in LRRK2-G2019S microglia has a neuroprotective effect, we assessed dopaminergic neuron survival in midbrain organoids cocultured with microglia and treated with or without oxamic acid. Compared with organoids cultured with healthy microglia, midbrain organoids cultured with LRRK2-G2019S microglia showed significant dopaminergic neuron loss (Fig. 6F). However, treatment with oxamic acid substantially increased the number of dopaminergic neurons in midbrain organoids cultured with LRRK2-G2019S microglia, ameliorating the detrimental impact of LRRK2-G2019S microglia. These data indicate that targeting microglia metabolism in LRRK2-G2019S microglia not only reduces their inflammatory phenotype but also prevents neurotoxicity, demonstrating the potential of immunometabolic approaches that may slow neurodegeneration, pending further validation.

## Discussion

The G2019S mutation in LRRK2 represents one of the most common causes of familial PD [41]. LRRK2 regulates a variety of cellular functions, including mitochondrial function, vesicle trafficking, autophagy, cell growth, differentiation, and metabolism [27]. Despite extensive research, the exact mechanism by which LRRK2 contributes to PD pathogenesis remains unclear. Notably, neuroinflammation has emerged as an important characteristic of PD. LRRK2 is also highly expressed in various immune cells, where its expression correlates with immune activity, suggesting a potential role in neuroinflammation and neurodegeneration associated with PD.

This study investigated the impact of the LRRK2-G2019S mutation on microglial function in human iPSCs-derived models. Specifically, we employed in vitro midbrain organoids to evaluate the effects of LRRK2-G2019S microglia on dopaminergic neuron integrity and PD pathogenesis. While previous studies have shown altered microglial activity in both murine and human iPSC-derived microglia [42–44], details on the molecular mechanism driving this altered activity are limited. In particular, the interaction between LRRK2-G2019S microglia and dopaminergic neurons in a representative brain organoid model has been underexplored.

Our results demonstrated for the first time that LRRK2-G2019S mutant microglia exhibit increased proinflammatory activities, which are associated with changes in their cellular metabolism. Proinflammatory microglia induces neurotoxicity and dopaminergic neuron loss in midbrain organoids. Importantly, restoring microglial function by targeting metabolic pathways prevents dopaminergic neuronal loss, suggesting a potential therapeutic avenue.

Our findings reveal that iPSC-derived microglia with the LRRK2-G2019S mutation exhibit increased expression of inflammatory markers and altered function, without changes in their differentiation or identity. Previous studies have indicated that LRRK2 expression increases upon stimulation with the TLR ligand LPS in bone marrow-derived macrophages and primary murine microglia [44, 45]. LRRK2 mutations in PD have also been shown to exacerbate inflammatory responses [46], while the loss of LRRK2 has been associated with decreased proinflammatory signaling and neuroprotective effects against LPS and α-synuclein-induced neurodegeneration [47]. TLR-driven neuroinflammation is linked to dopaminergic neuron loss in PD, and TLR modulation has been shown to reduce inflammation and improve PD symptoms [48]. Murine and human models harboring LRRK2 mutations resulted in significant increases in the levels of proinflammatory cytokines such as IFN-γ, TNF-α, IL-1α, IL-1β, IL-6, IL-8, IL-10, and CXCL-1, among many others [49, 50]. Interestingly, we observed exclusively the upregulation of TNF-α in microglia harboring LRRK2-G2019S mutation. TNFα is not only a key regulator of inflammatory responses, but also in certain circumstances can cause cell death by apoptosis and necroptosis [51]. In the CNS, TNFα is linked to neuronal loss, which is mediated by microglial phagocytosis, as well as neuronal necroptosis [52, 53], possibly a key driver of dopaminergic neuron degeneration in PD.

Pathway analysis highlighted altered ROS metabolism, the NRF2 antioxidant pathway, and mTOR signaling in LRRK2-G2019S microglia. Changes in ROS-associated pathways could be due to compensatory mechanism, as elevated metabolism will produce higher ROS levels, which need to be buffered in LRRK2-G2019S microglia. This is also reflected by unchanged cytoplasmic ROS nor mitochondrial ROS in LRRK2-G2019S microglia compared to WT microglia. Context-specific metabolic models predicted metabolic remodeling in these mutant microglia, linking immune dysfunction to cellular metabolic changes. Dysregulated metabolic pathways in immune cells contribute to a wide range of diseases from autoinflammatory diseases to cancers [10, 54, 55]. Altered metabolism likely drives increased inflammatory activity in LRRK2-G2019S microglia, a mechanism implicated in various diseases.

Our data showed mTOR upregulation in LRRK2-G2019S microglia. mTOR senses the intra- and extracellular nutrient status, growth factor, and cell stress-related changes to coordinate cellular metabolism and balance immunological functions [56, 57]. Cheng et al. demonstrated that glycolytic reprogramming controls microglial inflammatory activation, and that glycolysis inhibition reduces NF-κB transcriptional activity and neuroinflammation [58]. We observed upregulated mTOR shifted LRRK2-G2019S microglial metabolism toward glycolysis, driving microglial inflammation. In fact, on adipocytes, LRRK2 substrates RAB10 has been shown as a downstream target of AS160 and a positive regulator of GLUT4 trafficking to the cell surface upon insulin stimulation, where RAB10 knockdown led to twofold decrease in GLUT4 exocytosis rate [59]. Moreover, Panagiotakopoulou et al., also showed that LRRK2-G2019S mutation led to changes in cytokine production and glycolytic switch in NFAT-independent manner, further highlighting the close relation between LRRK2, microglia inflammation and metabolic remodeling [60].

We noted impaired serine synthesis in LRRK2-G2019S microglia with reduced expression of the key genes PHGDH and PSAT1. In addition, metabolic tracing of labelled ^13^C_6_ glucose confirmed decreased glucose-derived serine in LRRK2-G2019S microglia. Serine is a nonessential amino acid that play diverse physiological functions, including one carbon metabolism for nucleotide synthesis and epigenetic modification, as well as redox balance [61, 62]. Previously, serine deprivation was shown to impair mTOR signaling, IL-1β secretion and inflammasome activation [63]. In fact, altered serine metabolism between the conversion of L and D serine by serine racemase enzyme has also been explored in neuroepithelial stem cells harboring LRRK2-G2019S mutation [64]. Interestingly, LRRK2-G2019S microglia showed increased mTOR activity, shifting toward glycolysis, and heightened immune activation. This metabolic adaptation may exacerbate neuroinflammation and impair dopaminergic neuron health.

In 3D midbrain organoid models, the addition of LRRK2-G2019S microglia led to dopaminergic neuron loss compared with that in midbrain organoids with healthy microglia, highlighting the neurotoxic effect of LRRK2-G2019S mutant microglia on dopaminergic neurons. Treating these microglia with oxamic acid, a known mTOR and glycolysis inhibitor [39, 40], normalized their metabolism, reduced inflammation, and preserved their phagocytic function. Phagocytosis is known to be beneficial for clearing cellular debris and protein aggregates, thereby protecting the CNS microenvironment and neurons from stress [65]. The uncoupling of phagocytosis and inflammation by oxamic acid undoubtedly adds an extra dimension in ensuring normal cellular clearance and inflammatory activity by microglia. Oxamic acid notably lowered TNF-α levels, thus preventing dopaminergic neuronal loss in organoid models with LRRK2-G2019S microglia. These findings suggest inhibiting glucose metabolism in LRRK2-G2019S microglia could mitigate their TNF-α dependent neurotoxic effects on dopaminergic neurons, demonstrating its neuroprotective potential.

In summary, this study provides new insights into the role of the LRRK2-G2019S mutation in modulating microglial function and PD pathogenesis. LRRK2 mutations promote immune hyperactivation and metabolic alterations, contributing to neuroinflammation and dopaminergic neuronal degeneration in healthy midbrain organoids. Targeting microglial metabolism with oxamic acid reduces TNF-α-dependent neuroinflammation while maintaining normal phagocytic function, underscoring the ideal therapeutic potential for disease-modifying strategies in PD.

### Limitations

While our study provides novel insight into how LRRK2-G2019S microglia contribute to dopaminergic neuron degeneration through metabolic changes and inflammatory activation, several limitations should be considered. First, although we identified associations between glycolytic rewiring, impaired serine metabolism, increased inflammation, and neuronal vulnerability, our study does not establish direct causality between these processes. Whether metabolic reprogramming is the primary driver of inflammatory activation or a parallel consequence of LRRK2-G2019S signaling remains to be explored. Second, the altered pathways we identified such as ROS and mTOR signaling, may represent secondary or compensatory responses to the mutation rather than primary pathogenic mechanisms. Dissecting the temporal sequence of these changes will require more targeted perturbation or gene editing studies. Third, our analyses centered primarily on glycolysis and serine metabolism, but microglia also utilize broad metabolic networks including glutamine or lipid metabolism, which may also shape inflammatory phenotypes in LRRK2-G2019S context. A more comprehensive metabolic profiling, including isotope tracing and lipidomics, will be essential to fully capture the extent of metabolic vulnerabilities.

Moreover, while oxamic acid treatment was able to rescue aberrant microglial phenotypes and dopaminergic neuron loss in midbrain organoid, its off-target effects and translational feasibility remain to be validated in animal models. Thus, future studies employing alternative and more selective metabolic modulators will be required to confirm specificity and translational potential. Despite these limitations, our findings advance understanding of how LRRK2-G2019S mutations alter microglial metabolism and inflammatory response, and they underscore the potential of targeting immunometabolic pathways as a therapeutic avenue in PD.

## Supplementary Information

Supplementary material 1.

## Data Availability

All original and processed data as well as the scripts generated from the study are publicly available under this DOI: 10.17881/b3yf-xt27 **.** The bulk RNAseq gene expression dataset generated for this manuscript can be accessed in the Gene Expression Omnibus database under the identifier GSE282494.
