# Supplementary material for: The Parkinson’s disease-associated LRRK2-G2019S variant restricts serine metabolism, leading to microglial inflammation and dopaminergic neuron degeneration
Source: J Neuroinflammation. 2025 Oct 27;22:244. doi: 10.1186/s12974-025-03577-2 (PMC12560597; doi:10.1186/s12974-025-03577-2)
Supplement: Supplementary file 1 — Supplementary material 1. [file 12974_2025_3577_MOESM1_ESM.pdf]

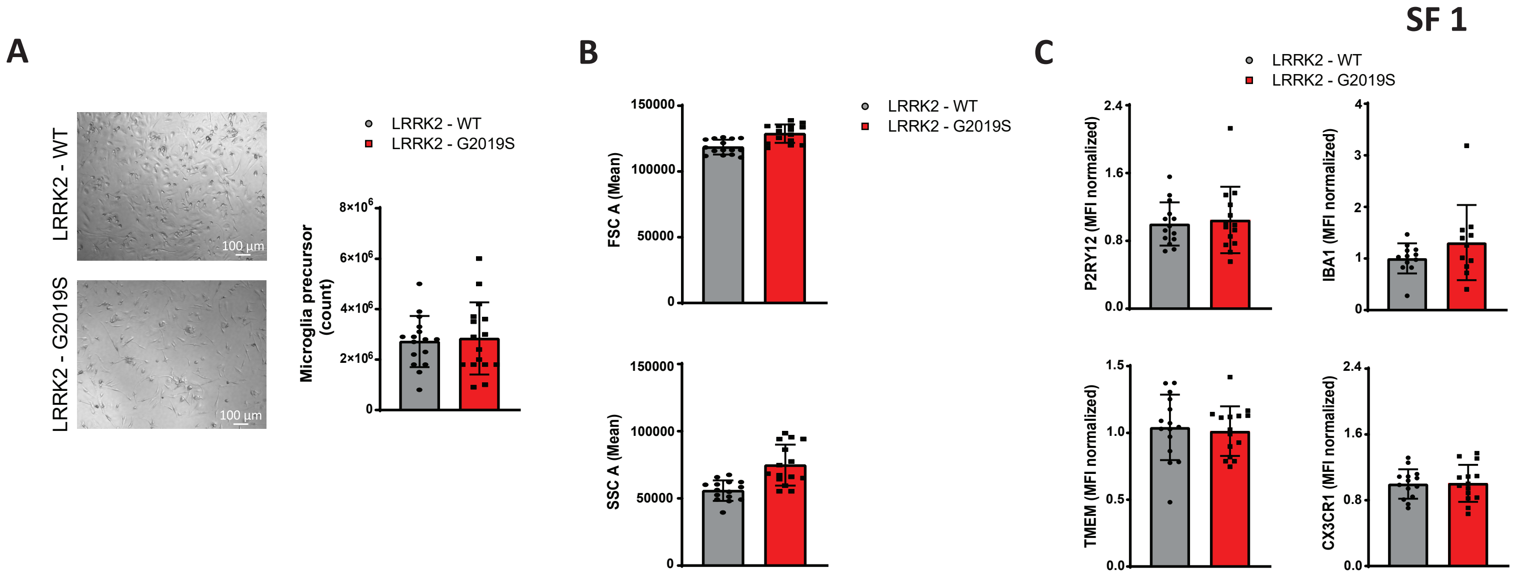
SF 1
A
LRRK2 - WT
LRRK2 - G2019S
100 µm
100 µm
LRRK2 - WT
LRRK2 - G2019S
Microglia precursor (count)
8×10^6
6×10^6
4×10^6
2×10^6
0
B
FSC A (Mean)
150000
100000
50000
0
SSC A (Mean)
LRRK2 - WT
LRRK2 - G2019S
C
LRRK2 - WT
LRRK2 - G2019S
P2RY12 (MFI normalized)
2.4
1.6
0.8
0.0
IBA1 (MFI normalized)
4
3
2
1
0
TMEM (MFI normalized)
1.5
1.0
0.5
0.0
CX3CR1 (MFI normalized)
2.4
1.6
0.8
0.0

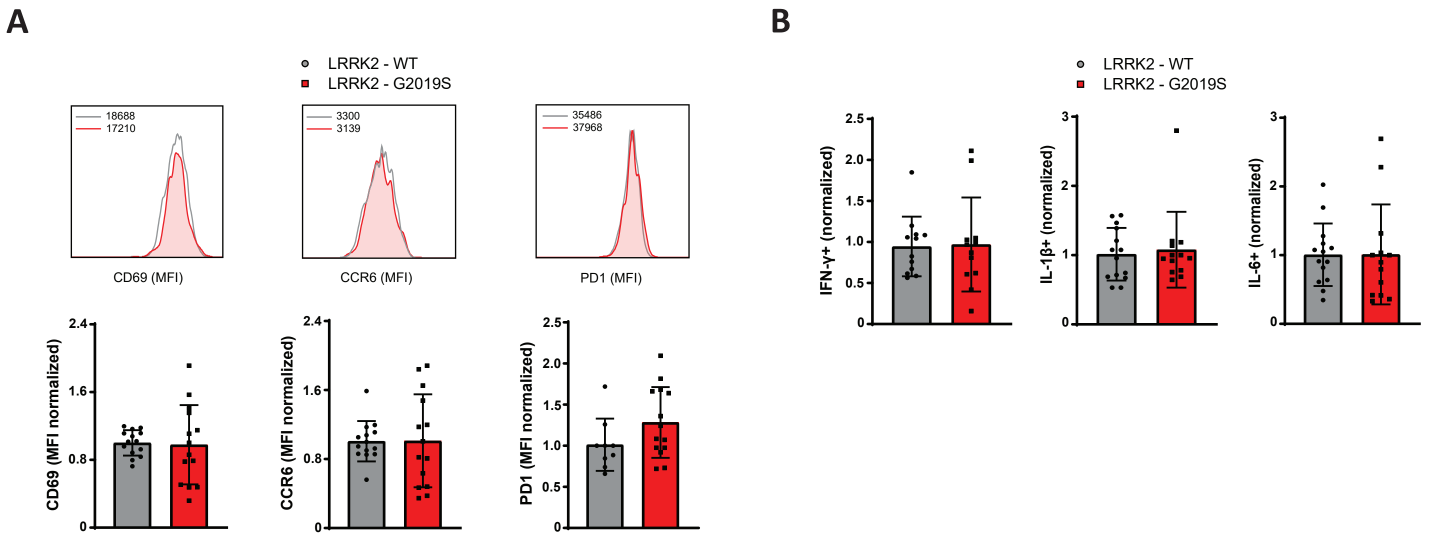
A
LRRK2 - WT
LRRK2 - G2019S
18688
17210
CD69 (MFI)
3300
3139
CCR6 (MFI)
35486
37968
PD1 (MFI)
CD69 (MFI normalized)
CCR6 (MFI normalized)
PD1 (MFI normalized)
B
LRRK2 - WT
LRRK2 - G2019S
IFN-γ+ (normalized)
IL-1β+ (normalized)
IL-6+ (normalized)

SF 3

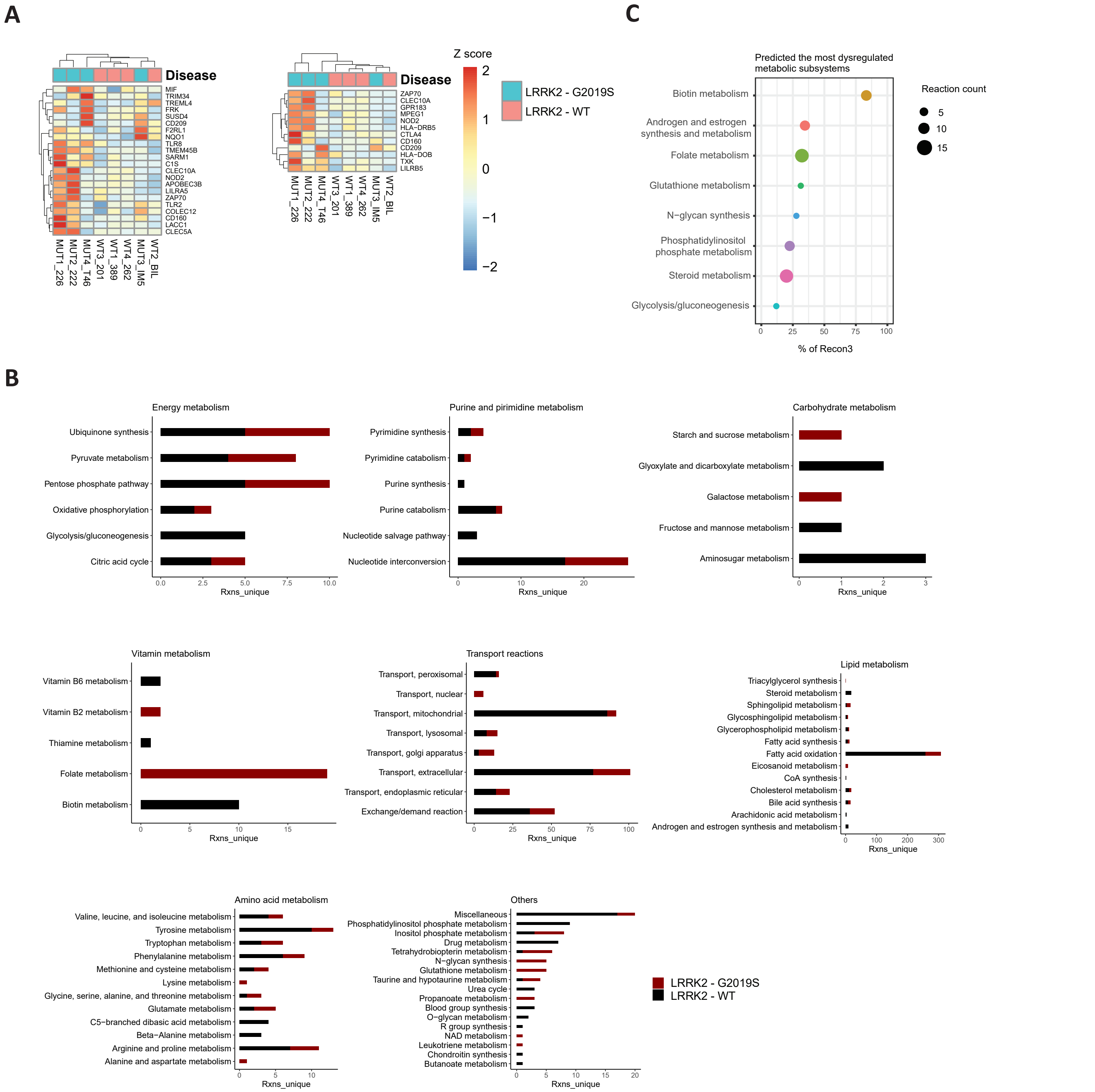

A
Disease
MIF
TRIM34
TREML4
FRK
SUSD4
CD209
F2RL1
NQO1
TLR8
TMEM45B
SARM1
C1S
CLEC10A
NOD2
APOBEC3B
LILRA5
ZAP70
TLR2
COLEC12
CD160
LACC1
CLEC5A
MUT1_226
MUT2_222
MUT4_T46
WT3_201
WT1_389
WT4_262
MUT3_IM5
WT2_BIL
Disease
ZAP70
CLEC10A
GPR183
MPEG1
NOD2
HLA−DRB5
CTLA4
CD160
CD209
HLA−DOB
TXK
LILRB5
MUT1_226
MUT2_222
MUT4_T46
WT3_201
WT1_389
WT4_262
MUT3_IM5
WT2_BIL
Z score
2
1
0
−1
−2
LRRK2 - G2019S
LRRK2 - WT
C
Predicted the most dysregulated metabolic subsystems
Biotin metabolism
Androgen and estrogen synthesis and metabolism
Folate metabolism
Glutathione metabolism
N−glycan synthesis
Phosphatidylinositol phosphate metabolism
Steroid metabolism
Glycolysis/gluconeogenesis
0
25
50
75
100
% of Recon3
Reaction count
5
10
15
B
Energy metabolism
Ubiquinone synthesis
Pyruvate metabolism
Pentose phosphate pathway
Oxidative phosphorylation
Glycolysis/gluconeogenesis
Citric acid cycle
0.0
2.5
5.0
7.5
10.0
Rxns_unique
Purine and pirimidine metabolism
Pyrimidine synthesis
Pyrimidine catabolism
Purine synthesis
Purine catabolism
Nucleotide salvage pathway
Nucleotide interconversion
0
10
20
Rxns_unique
Carbohydrate metabolism
Starch and sucrose metabolism
Glyoxylate and dicarboxylate metabolism
Galactose metabolism
Fructose and mannose metabolism
Aminosugar metabolism
0
1
2
3
Rxns_unique
Vitamin metabolism
Vitamin B6 metabolism
Vitamin B2 metabolism
Thiamine metabolism
Folate metabolism
Biotin metabolism
0
5
10
15
Rxns_unique
Transport reactions
Transport, peroxisomal
Transport, nuclear
Transport, mitochondrial
Transport, lysosomal
Transport, golgi apparatus
Transport, extracellular
Transport, endoplasmic reticular
Exchange/demand reaction
0
25
50
75
100
Rxns_unique
Lipid metabolism
Triacylglycerol synthesis
Steroid metabolism
Sphingolipid metabolism
Glycosphingolipid metabolism
Glycerophospholipid metabolism
Fatty acid synthesis
Fatty acid oxidation
Eicosanoid metabolism
CoA synthesis
Cholesterol metabolism
Bile acid synthesis
Arachidonic acid metabolism
Androgen and estrogen synthesis and metabolism
0
100
200
300
Rxns_unique
Amino acid metabolism
Valine, leucine, and isoleucine metabolism
Tyrosine metabolism
Tryptophan metabolism
Phenylalanine metabolism
Methionine and cysteine metabolism
Lysine metabolism
Glycine, serine, alanine, and threonine metabolism
Glutamate metabolism
C5−branched dibasic acid metabolism
Beta−Alanine metabolism
Arginine and proline metabolism
Alanine and aspartate metabolism
0
5
10
Rxns_unique
Others
Miscellaneous
Phosphatidylinositol phosphate metabolism
Inositol phosphate metabolism
Drug metabolism
Tetrahydrobiopterin metabolism
N−glycan synthesis
Glutathione metabolism
Taurine and hypotaurine metabolism
Urea cycle
Propanoate metabolism
Blood group synthesis
O−glycan metabolism
R group synthesis
NAD metabolism
Leukotriene metabolism
Chondroitin synthesis
Butanoate metabolism
0
5
10
15
20
Rxns_unique
LRRK2 - G2019S
LRRK2 - WT

SF 4

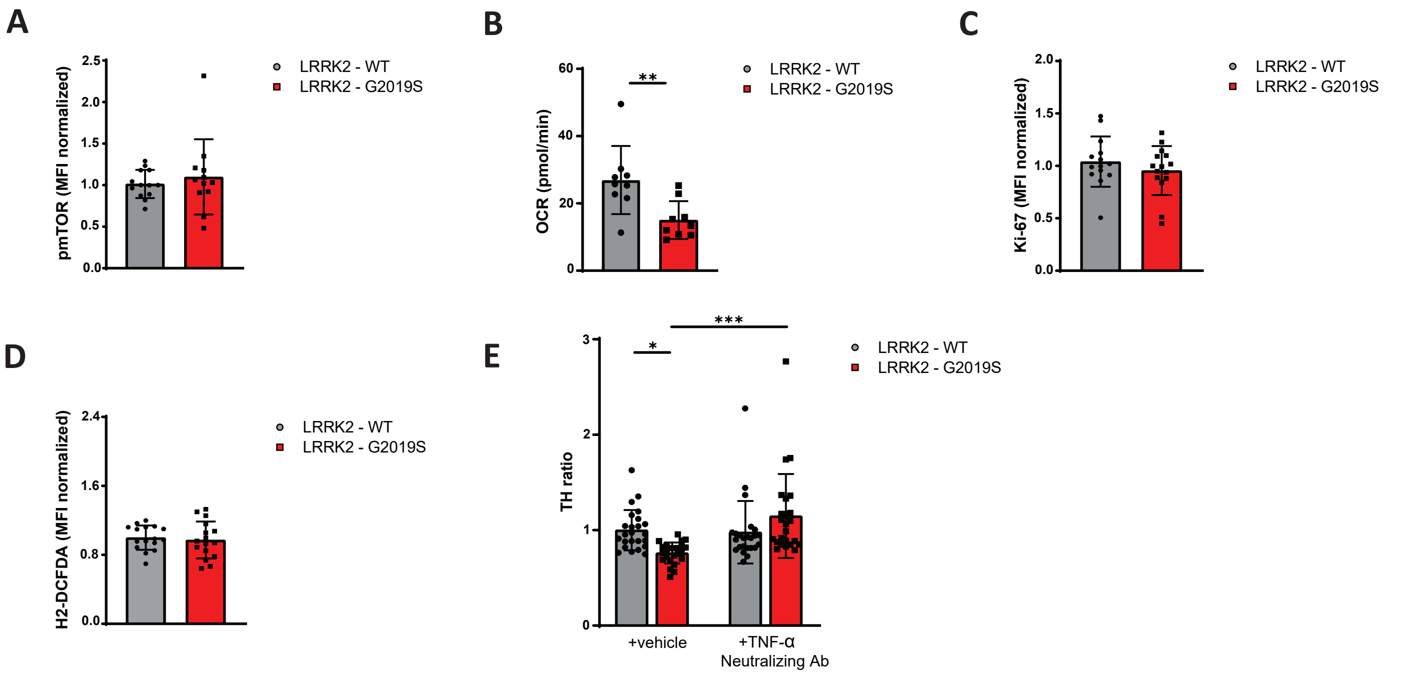
A
pmTOR (MFI normalized)
2.5
2.0
1.5
1.0
0.5
0.0
LRRK2 - WT
LRRK2 - G2019S
B
OCR (pmol/min)
60
40
20
0
**
LRRK2 - WT
LRRK2 - G2019S
C
Ki-67 (MFI normalized)
2.0
1.5
1.0
0.5
0.0
LRRK2 - WT
LRRK2 - G2019S
D
H2-DCFDA (MFI normalized)
2.4
1.6
0.8
0.0
LRRK2 - WT
LRRK2 - G2019S
E
TH ratio
3
2
1
0
*
***
+vehicle
+TNF-α
Neutralizing Ab
LRRK2 - WT
LRRK2 - G2019S

Supplementary Figure Legends

Figure SF1: Similar properties of iPSC-derived microglia harboring the LRRK2-G2019S mutation. (A) Brightfield images of microglia differentiated from LRRK2-WT and LRRK2-G2019S iPSCs (left). Quantification of macrophage precursors from LRRK2-WT and LRRK2-G2019S iPSCs (right). Data are shown as mean ± SD (n = 3-4), pooled from three independent trials. (B) Flow cytometry analysis of morphological features of LRRK2-WT and LRRK2-G2019S microglia, showing forward scatter area (size, top) and side scatter area (complexity, bottom). Data are shown as mean ± SD (n = 3-4), pooled from three independent trials. (C) Quantification of mean fluorescence intensity of identity markers (P2RY12, IBA1, TMEM119, CX3CR1) of mature LRRK2-WT vs LRRK2-G2019S microglia using flow cytometry. Data are shown as mean ± SD (n = 3-4), pooled from three independent trials.

Figure SF2: Similar expression of microglia associated markers and pro-inflammatory cytokines in LRRK2-G2019S microglia. (A) Representative analysis (top) and quantification of mean fluorescence intensity (bottom) of markers (CD69, CCR6, PD1) of LRRK2-WT vs LRRK2-G2019S microglia using flow cytometry. Data are shown as mean ± SD (n = 3-4), pooled from three independent trials. (B) Flow cytometry analysis of intracellular cytokine IFN-γ, IL-1β, and IL-6 in LRRK2-WT and LRRK2-G2019S microglia after 24h stimulation with LPS. Data are shown as mean ± SD (n = 3-4), pooled from three independent trials.

Figure SF3: Systemic changes of metabolic pathways based on the metabolic modelling in LRRK2-G2019S microglia. (A) Heatmap of innate immunity genes (left) and adaptive immunity genes (right) differentiating LRRK2-WT and LRRK2-G2019S microglia upon hierarchical clustering with FC>0.5. (B) Comparison of LRRK2-WT and LRRK20G2019S microglia metabolic model composition. Reactions found exclusively in LRRK2-WT microglia (black) or in LRRK2-G2019S microglia (red) were assigned to corresponding Recon 3 subsystems. (C) The most predicted dysregulated metabolic subsystems between LRRK2-WT and LRRK2-G2019S microglia after pooling the exclusive reactions from both conditions. Dot size and color indicate the number of reactions per subsystem. The location on y axis indicates how many reactions of the total subsystems in Recon 3 are affected.

Figure SF4: Similar upstream mTOR activity and proliferative capacity in LRRK2-G2019S microglia. (A) Flow cytometry analysis of mTOR phosphorylation in LRRK2-WT and LRRK2-G2019S microglia. Data are shown as mean ± SD (n = 3-4), pooled from three independent trials. (B) Measurement of oxygen consumption rate (OCR) in LRRK2-WT and LRRK2-G2019S microglia using the Seahorse Cell Mitochondrial Stress Test. Proton leak was quantified. Data are shown as mean ± SD (n = 3-4), pooled from three independent trials. (C) Flow cytometry analysis of intracellular protein Ki-67 in LRRK2-WT and LRRK2-G2019S microglia. Data are shown as mean ± SD (n = 3-4), pooled from three independent trials. (D) Flow cytometry analysis of reactive oxygen species levels using H2-DCFDA in LRRK2-WT and LRRK2-G2019S microglia. Data are shown as mean ± SD (n = 3-4), pooled from three independent trials. (E) The ratio of dopaminergic neurons, represented by the total TH+ cells over total MAP2+ neurons, in midbrain organoids treated with or without 1 ng/ml TNF-α neutralizing antibody. Data are shown as mean ± SD (n = 3), pooled from three independent trials.

Supplementary Table 1: List of materials (antibody, small molecules, probes, and commercial kits) used in this study.

REAGENT or RESOURCE	SOURCE	Cat#	RRIDS
**Antibodies**			
PU.1	Cell signalling	2258S	AB_2186909
IBA1	Abcam	Ab5076	AB_2224402
CD11b	Biolegend	101206	AB_312789
CD45	Biolegend	304007	AB_314395
P2RY12	Biolegend	392106	AB_2783921
TMEM119	Abcam	Ab225497	AB_3665680
CX3CR1	Biolegend	341627	AB_2810534
CD68	Biolegend	333822	AB_2571965
HLA-DR	Biolegend	307606	AB_314684
CD80	Biolegend	305218	AB_2076148
CD86	Biolegend	374214	AB_2734430
CD282	Biolegend	148604	AB_2564120
CD284	Biolegend	312816	AB_2562487
TNF-α	Biolegend	506344	AB_2565953
RPS6 phospho (Ser244)	Biolegend	935706	AB_3665682
PHGDH	Cell signalling	66350S	AB_2737030
TH	Abcam	Ab112	AB_297840
MAP2	Abcam	Ab92434	AB_2138147
IBA1	Abcam	Ab5076	AB_2224402
CD69	Biolegend	310904	AB_314839
CD196	Biolegend	353406	AB_10918437
CD279	Biolegend	329916	AB_2283437
IFN-γ	Biolegend	506528	AB_2566187
IL-6	Biolegend	501120	AB_2572042
IL-1β	Biolegend	508208	AB_604135
Phospho-mTOR (Ser2448)	Biolegend	48-9718-42	AB_2574127
Ki-67	Biolegend	350504	AB_10660752

donkey anti-goat Alexa Fluor ®488	Invitrogen	A11057	AB_142581
donkey anti-rabbit Alexa Fluor ®568	Invitrogen	A10042	AB_2534017
goat anti-rabbit Alexa Fluor ®488	Invitrogen	A11034	AB_2576217
goat anti-mouse Alexa Fluor®568	Invitrogen	A11031	AB_144696
goat anti-chicken Alexa Fluor®647	Invitrogen	A-21449	AB_2535866
goat anti-mouse Alexa Fluor ®488	Invitrogen	A-11001	AB_2534069
goat anti-rabbit Alexa Fluor ®647	Invitrogen	A-21244	AB_2535812
**Chemicals, Peptides, and Recombinant Proteins**			
MitoTracker™ Green FM	ThermoFisher Scientific	M7514	
MitoTracker™ Deep Red FM	ThermoFisher Scientific	M22426	
H2DCFDA	ThermoFisher Scientific	D399	
Zombie Green™ Fixable Viability Kit	Biolegend	423112	
Zombie NIR™ Fixable Viability Kit	Biolegend	423106	
2-(N-(7-Nitrobenz-2-oxa-1,3-diazol-4-yl)Amino)-2-Deoxyglucose	ThermoFisher Scientific	N13195	
Hoechst33342	Thermo Fisher Scientific	62249	
Zymosan A S.cerevisiae BioParticles™	ThermoFisher Scientific	Z2841	
pHrodo™ Red Zymosan BioParticles™	ThermoFisher Scientific	P35364	
Oligomycin A	Sigma-Aldrich	75351-5MG	
FCCP	Sigma-Aldrich	C2920-10MG	
Antimycin A	Sigma-Aldrich	A8674-25MG	
Rotenone	Sigma-Aldrich	R8875-1G	
D-Glucose (U-$^{13}C_6$, 99%)	Cambridge Isotope Laboratories, Inc	CLM-1396-1	
D-Glucose	Sigma-Aldrich	G8270-5KG	
2-Deoxy-D-glucose	Sigma-Aldrich	D6134-1G	
EDTA	Sigma-Aldrich	E9884-100G	
Saponin	Sigma-Aldrich	S4521-25G	
Formaldehyde	Sigma-Aldrich	252549-1L	
HEPES	Sigma-Aldrich	H4034-100G	
Methanol	Sigma-Aldrich	1060351000	

Chloroform	Sigma-Aldrich	34584-1L-M	
GolgiPlug™	BD Biosciences	555029	
Corning™ Cell-Tak Cell and Tissue Adhesive	ThermoFisher Scientific	10317081	
Sodium pyruvate	GIBCO	12539059	
2-mercaptoethanol	GIBCO	11508916	
Penicillin/Streptomycin	ThermoFisher Scientific	15140122	
DMEM/F12 Advanced medium	ThermoFisher Scientific	12634010	
Essential 8	ThermoFisher Scientific	A1517001	
DMEM/F12 (1:1) W/O L-GLUT	ThermoFisher Scientific	21331046	
Neurobasal medium	ThermoFisher Scientific	21103049	
B27 – vitamin A	ThermoFisher Scientific	12587001	
GlutaMAX	ThermoFisher Scientific	35050061	
Human IL-34 Recombinant Protein	Biolegend	577906	
Human GM-CSF Recombinant Protein	Biolegend	572905	
Human IL-3 Recombinant Protein	Biolegend	578008	
Human M-CSF Recombinant Protein	Biolegend	574808	
Human BMP-4 Recombinant Protein	Biolegend	795606	
Human VEGF-165 Recombinant Protein	Biolegend	583708	
Human SCF	Miltenyi Biotec	130-096-695	
BIOFLOAT™ 96 well plate U-bottom	Facellitate	F202003	
Aggrewell 800 24 well plate	StemCell technologies	34815	
L-Ascorbic acid	Sigma-Aldrich	A4544-100G	
SB431542	Sigma-Aldrich	ab120163	
CHIR99021	Axon	CT 99021	
Smoothened agonist	Merck	566660-1MG	
LDN193189	Sigma-Aldrich	SML0559-5MG	
ROCK inhibitor Y-27632 2HCl [146986-50-7, 129830-38-2]	CliniSciences	A11001-50	

pluriStrainer® 40 µm (Cell Strainer)	PluriSelect	SKU 43-50040-51	
N2 supplement 100x 50ml	ThermoFisher Scientific	17502001	
Recombinant Human GDNF	Peprotech	450-10-1mg	
Recombinant Human BDNF	Peprotech	450-02 - 1mg	
DAPT	R&D Systems	2634/50	
Human ActivinA Recombinant Protein	ThermoFisher Scientific	PHC9561	
X-VIVO 15 with Gentamicin and Phenol Red, 1 L	Lonza	LO BE02-060Q	
**Critical Commercial Assays**			
RNeasy kit	Qiagen	74106	
Human Inflammatory Cytokine Multiplex ELISA Kit	Arigo Laboratories	ARG80929	
CellTiter-Glo® Luminescent Cell Viability Assay	Promega	G7571	
Seahorse XFe96 Fluxpak	Agilent Technologies	102416-100	
BD Pharmingen™ Transcription Factor Buffer Set	BD Biosciences	554656	AB_2869424
**Software and Algorithms**			
R			
Graphpad Prism			
Matlab			
Image J			
Seahorse Wave			
FlowJo			
Zen blue/black			
